# Urban occupational structures as information networks: The effect on network density of increasing number of occupations

**DOI:** 10.1371/journal.pone.0196915

**Published:** 2018-05-07

**Authors:** Shade T. Shutters, José Lobo, Rachata Muneepeerakul, Deborah Strumsky, Charlotta Mellander, Matthias Brachert, Teresa Farinha, Luis M. A. Bettencourt

**Affiliations:** 1 Global Security Initiative, Arizona State University, Tempe, Arizona, United States of America; 2 School of Sustainability, Arizona State University, Tempe, Arizona, United States of America; 3 Department of Agricultural and Biological Engineering, University of Florida, Gainesville, Florida, United States of America; 4 Arizona State University-Santa Fe Institute Center for Biosocial Complex Systems, Arizona State University, Tempe, Arizona, United States of America; 5 Department of Economics, Jönköping International Business School, Jönköping University, Jönköping, Sweden; 6 Department of Structural Change and Productivity, Halle Institute for Economic Research, Halle (Saale), Germany; 7 Department of Economic Geography, Human Geography and Spatial Planning, Utrecht University, Utrecht, Netherlands; 8 IN+ Center for Innovation, Technology and Policy Research, Universidade de Lisboa, Lisboa, Portugal; 9 Santa Fe Institute, Santa Fe, New Mexico, United States of America; Iowa State University, UNITED STATES

## Abstract

Urban economies are composed of diverse activities, embodied in labor occupations, which depend on one another to produce goods and services. Yet little is known about how the nature and intensity of these interdependences change as cities increase in population size and economic complexity. Understanding the relationship between occupational interdependencies and the number of occupations defining an urban economy is relevant because interdependence within a networked system has implications for system resilience and for how easily can the structure of the network be modified. Here, we represent the interdependencies among occupations in a city as a non-spatial information network, where the strengths of interdependence between pairs of occupations determine the strengths of the links in the network. Using those quantified link strengths we calculate a single metric of interdependence–or connectedness–which is equivalent to the density of a city’s weighted occupational network. We then examine urban systems in six industrialized countries, analyzing how the density of urban occupational networks changes with network size, measured as the number of unique occupations present in an urban workforce. We find that in all six countries, density, or economic interdependence, increases superlinearly with the number of distinct occupations. Because connections among occupations represent flows of information, we provide evidence that connectivity scales superlinearly with network size in information networks.

## Introduction

Urban economies are intricate webs of exchange, linking specialized production units and manifesting divisions of labor and knowledge flows [1–5]. The specific goods and services such units can provide, and how well they provide them, are largely determined by the technologies, skills, and tacit knowledge integrated in the process of value creation. The interconnections among these technologies and skills form an economic structure—a structure that enables some developmental pathways while foreclosing others. A city’s transition from one type of economy to another must ultimately alter its current underlying economic structure by breaking down some parts and building up others. Thus, it is critical to develop a comprehensive understanding of the properties and dynamics of the structures underlying urban economies.

The connections in an economic network represent acts of exchange involving the flow of capital, the transportation of goods, the movement of people or, importantly, the transmission of information [6]. It is the exchange and flow of information, mediated by economic markets as well as by other channels, that makes it possible to coordinate activities, generate complementarities, and self-organize production and consumption [7, 8]. These lines of reasoning underpin the expectation that the productivity of an economy (national or urban) should increase as its level of connectivity among its constituent units increases. Under “network effects” the value of a product or service is dependent on the number of others using it implying increasing returns to network size [9], where size is the number of individual users. A similar theme runs through other networked processes. “Metcalfe’s Law” [10], “Reed’s Law” [11], and “Beckstrom’s Law” [12, 13], all posit that the utility or value of a network increases faster than linearly with network size, where size is the number of noes in the network. The sharing, matching, and learning mechanisms theorized as the underpinnings of urban agglomeration economies also represent instances in which larger, and more connected, networks of economic agents generate positive externalities [14].

How can the structure of an urban economy be conceptualized and analyzed? The degree to which a city can change its economic structure is determined in part by the city’s current pool of technologies and skills [15–19]. Because labor occupations are defined on the basis of skills and manipulation of technologies [20], the occupations prevalent in a city are a direct indicator of the city’s current skills and technological capabilities and are thus almost ideal observational units with which to define the city’s economic structure. The structure of an urban economy can be thought of as a set of occupations and the interconnections between those occupations. In other words, the structure of an urban economy is manifested by its network of occupations. Recent work has applied this network perspective to explore how cities transform their economies by altering their occupational network structure [21–23]. As with any network, a crucial feature of an urban occupational network is the nature and density of the links, or interdependencies, between occupations.

The regularities exhibited by the relationship among the number and diversity of occupations and population size in urban areas has long been studied by urban economics, economic geography and regional science [24–30] and have been recently revisited under a complexity science perspective [31–33]. The common empirical thrust of all these investigations is that larger urban areas (with respect to population or workforce size) sustain a larger number of distinct occupations than smaller sized ones. Here we examine how urban scale, measured as the number of distinct occupations, affects the connectivity among these occupations. What we seek to elucidate here is whether, in an information-rich milieu characterized by greater diversity of skills, the likely intensity of interconnectivity among these occupations increases systematically.

Availing ourselves of detailed urban occupational data for six well-established urban systems—those of the United States, Canada, Sweden, Portugal, Australia, and Germany, representing the most advanced production technologies—we construct urban occupational networks. Across those six countries we then compare the density (see definition in Materials & Methods) of a given city’s occupational network to its network size, where the network size is equal to the number of occupations or nodes in the network. These networks are constructed such that each node is a distinct occupation present in a given city and the links between nodes reflect the degree to which two occupations are interdependent [21]. The occupational networks we construct are weighted networks, meaning that we do not merely indicate the existence of a link between occupations, but quantify its intensity (i.e. weight).

The scaling relationship between urban occupations and urban size is of interest not only because of the socioeconomic importance of urban economies. Urban occupational networks are informational networks and their study provides insights into how such networks behave as the number of distinct nodes increases. It has become a common expectation from studying human agglomerations that increasing the scale of such agglomerations (whether they be hunter-gatherer groups, the first market economies, or modern cities) should facilitate a division of labor and the generation of new knowledge through the combination of existing ideas [34–36]. Studying urban occupational networks grant us the opportunity to quantify how the connectivity of an information network changes with the scale of the network.

### Modeling and estimation frameworks

The general advantages of connectivity in networked informational systems are premised on the division and coordination of labor and knowledge [6]. An urban occupational network reflects the division of labor that defines a particular urban economy, and the links connecting occupations reflect specific solutions to the coordination problem inherent in the production of goods and services. Some of the connections among occupations are necessary complementarities without which specialized occupations cannot fulfill specific tasks. But other types of connections among occupations might reflect interactions formed in order to realize new tasks or produce novel goods or services. Economic innovation might result from the opportunities presented by interactions among a growing set of occupations [37]. Other apparent complementarities may result simply from a deeper division of knowledge in larger networks across organizations and places.

How should one expect the density of connections in urban occupational network to scale with the number of occupations? Suppose that urban occupation networks are indeed not simply about satisfying infrastructural needs or meeting input needs, but also about exchange of information and integration of knowledge. The information flow in such a network can be thought of as an irreversible exchange so that the generation and transportation costs associated with connectivity can in turn be understood as dissipative in nature—as it is transmitted and processed the information gets transformed—and dependent on the intensity of the exchange. Even the most basic of social information exchanges, that between two individuals talking, entails a cost: the generation of information is not energy-free, nor is its transmission even if by direct oral means. Assuming that there is a minimal cost (thermodynamic and pecuniary) which must be borne to generate and transmit information, the various process involved in such generation and transmission may have a general expected cost per connection that is independent of the system size [6]. Here “connection” refers to physical infrastructure through which information is transmitted between two agents.

It can also be reasonably posited that the average connectivity cost per node is proportional to the number of connections (representing information infrastructure) and therefore to the size of the network. Considerations of network economics and agglomeration economics lead to the expectation that the productivity of any one activity (or occupation) should be proportional to average socioeconomic connectivity [38]. Whether it gets cheaper or more expensive to add a connection partly depends on technological and regulatory considerations. But if the benefits of network connectivity outdistance the cost of establishing connectivity, then increasing network scale should lead to increasing connectivity with the increase being greater than proportional.

We adopt power-law function to represent the relationship between the generalized density of an urban occupational network *D* and the size of the network measured by the number of distinct occupations *N*:

$$D_{i}=\alpha N_{i}^{\beta },$$ (1)

with *α* a prefactor capturing the effects of technology and institutional arrangements on the relationship between network size and connectivity, and the subscript *i* identifying time in a city. Note that *D* is the generalized definition of network density, which applies to weighted networks as well as unweighted networks, and is defined further in the Methods section. The choice of a power-law function assumes that the effect on connectivity of increasing network size is not additive but multiplicative which is to say that the increase in connectivity is driven by the interaction of many factors observationally summarized in an increase in network size [39]. The value of *β* can be estimated by transforming Eq (1) into a liner equation and regressing the natural logarithm of the measure of network connectivity on the logarithm of network size.

The value of the exponent *β* (an elasticity) determines how the connectivity of an urban occupational network varies with network size. Are urban occupation networks primarily about infrastructural and input complementarities or do they also represent the flow of information leading to new economic niches? If the former is the case, then the value of *β* should be approximately one, while if the increase in connectivity is driven by the flow of information and the greater scale-dependent opportunities available for creating new economic opportunities then *β* should be greater than one.

## Results

**Fig 1:**
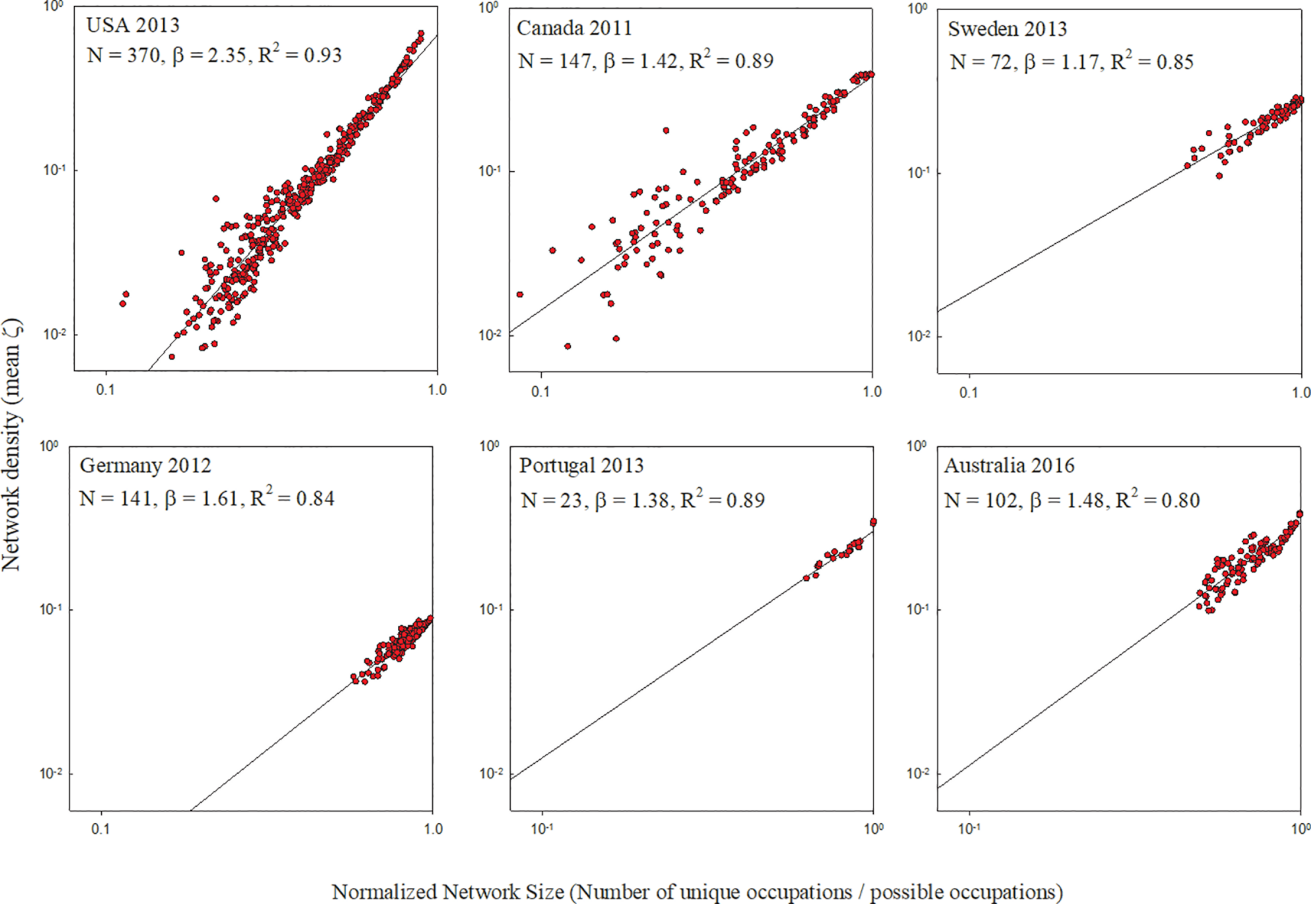
Network density versus network size. Among cities in the six countries studied, the density of a city’s occupational network increases superlinearly with the network’s size, measured as the number of unique occupations within the city. The exponent of a power law function for each country is given as β. Note that, for comparability, network size has been normalized by maximum possible size.

Using employment data from six industrialized countries, we first created an occupational network for every metropolitan area in each of those countries. We then measured both the size and the density, or mean link weight, of each occupational network. Our findings reveal, in all countries, a superlinear relationship between the size of a city’s occupational network and the density of that network (Fig 1), with the scaling exponent ranging from a high of 2.35 for U.S. cities to a low of 1.17 for Swedish cities (Table 1).

**Table 1:** Regression results (model *D* = α*N*^β^ where *D* = network density and *N* = number of unique occupations).

Country (Year)	No. Cities	No. Occupations (*N*)	Exponent (β)	95% C.I.	R^2^	p
USA (2013)	370	812	2.35	± 0.065	0.93	*
Canada (2011)	147	500	1.43	± 0.083	0.89	*
Germany (2012)	141	700	1.61	± 0.105	0.84	*
Australia (2016)	101	475	1.48	± 0.149	0.80	*
Sweden (2013)	72	355	1.17	± 0.116	0.85	*
Portugal (2013)	23	641	1.38	± 0.223	0.89	*

*—less than 0.0001

In all cases, the estimated scaling exponents are distinguishable from their trivial values (e.g. *β* = 1 or 0), in the absence of agglomeration effects, at 95% level of confidence (Tables 1 and 2). In all cases the superlinear relationship was significant (p < 0.00001), with R-square values ranging from 0.80 to 0.93.

**Table 2:** Supplemental regression results (model *D* = α*N*^β^ where *D* = network density and *N* = number of unique occupations). See Figs 3 and 4.

Country (Year)	No. Cities	No. Occupations (*N*)	Exponent (β)	95% C.I.	R^2^	p
USA (2013)	370	812	2.35	± 0.065	0.93	*
	370	455	2.36	± 0.072	0.92	*
	370	107	2.69	± 0.135	0.81	*
Germany (2012)	258	700	1.60	± 0.105	0.78	*
	141	700	1.61	± 0.116	0.84	*
	96	700	1.54	± 0.260	0.59	*

*—less than 0.0001

These results are based on a p-test level of confidence based on the value of the variance of the coefficient obtained by standard regression. Other methods have been proposed to estimate this variance—and associated level of confidence in measures of spatial sorting—based on null models of urns, for a small number of different types. Note however that a random assignment of types to locations of various sizes is different from this situation where locations may be otherwise similar but display different type compositions. In our case, statistical significance associated with the difference of exponents from proportional scaling provides us with the appropriate test.

**Fig 2:**
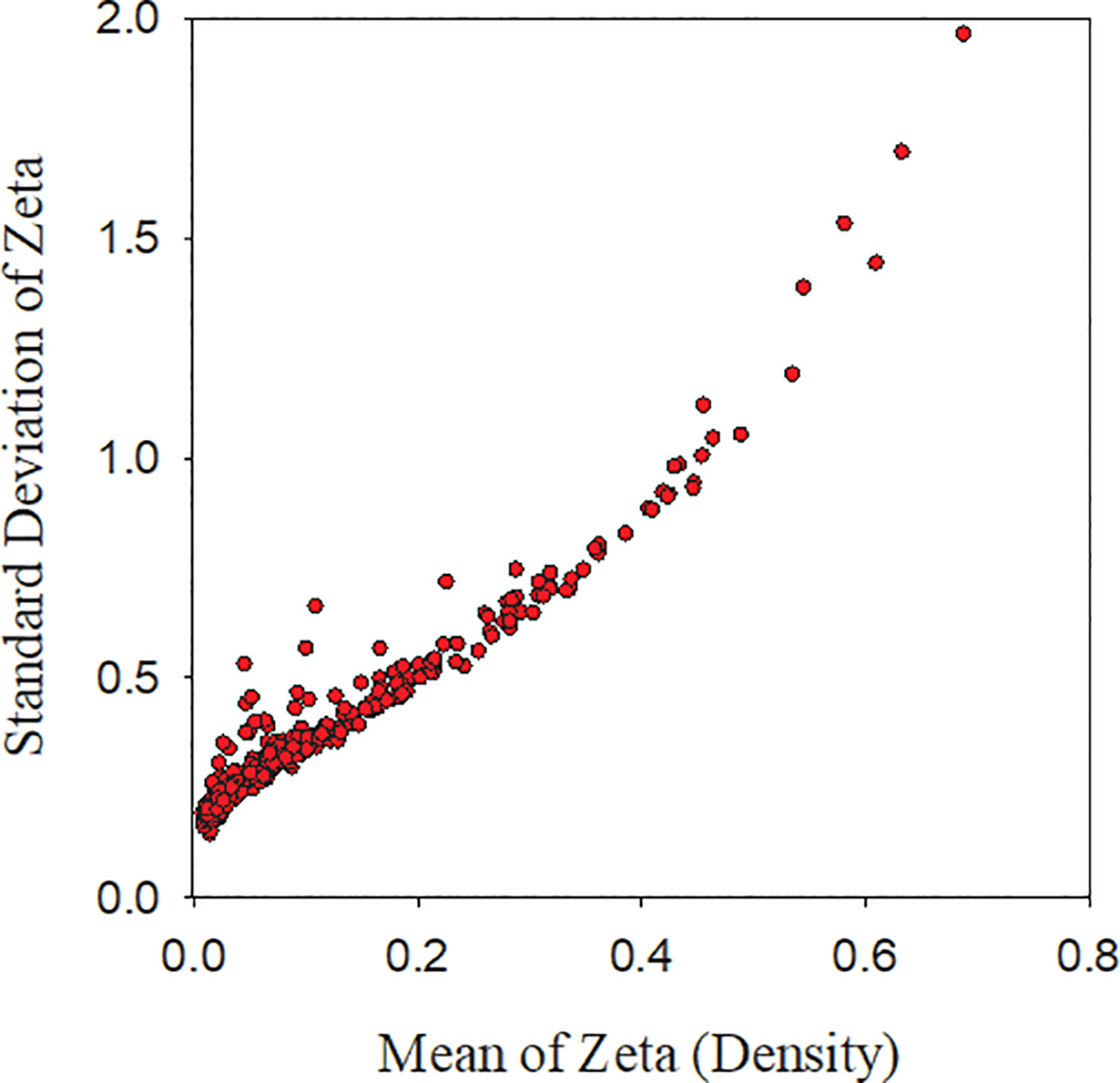
Network density vs. standard deviation of zeta for U.S. metropolitan areas. Increasing density (mean zeta) is correlated with increasing standard deviation of zeta driven by the appearance of rare and highly interdependent pairs of occupations.

Further, we find that the standard deviation of link values also increases with network size (Fig 2), albeit linearly.

**Fig 3:**
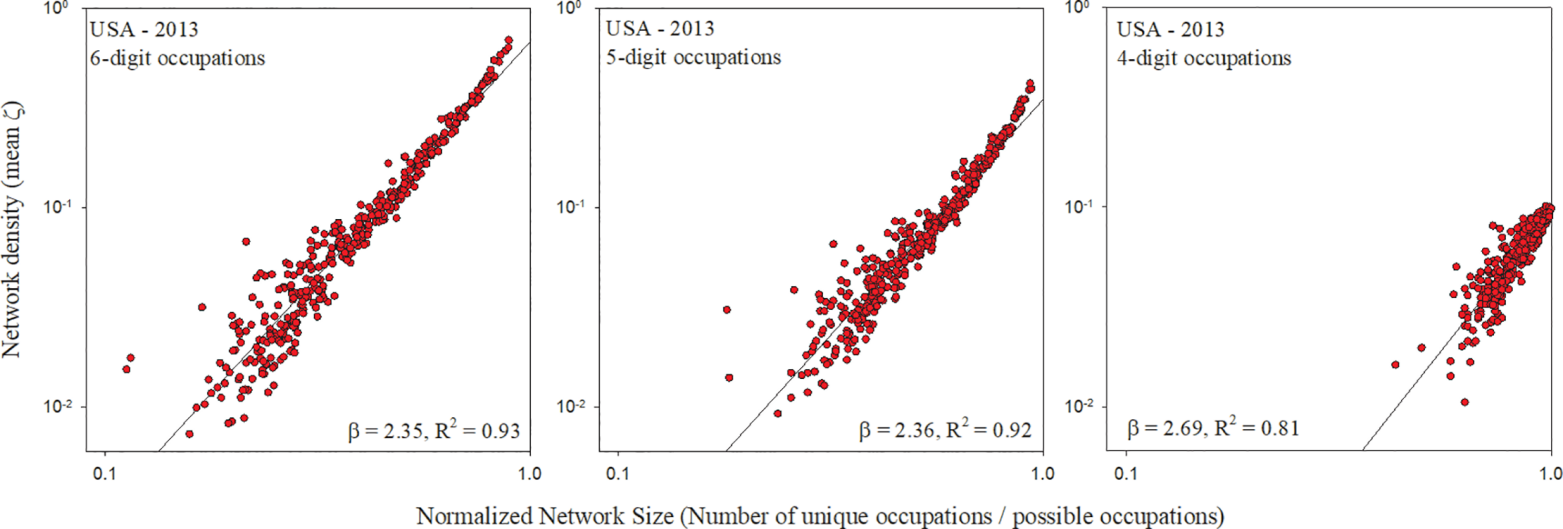
Occupational network size vs. density for U.S. cities at different employment aggregation levels. When occupational network size is compared to its density, the resulting scaling exponents differed little when 2013 U.S. employment is aggregated at the 6-digit, 5-digit, or 4-digit occupation code. The number of distinct occupations in each case are 812 (6-digit), 455 (5-digit), and 107 (4-digit). Note that, for comparability, network size has been normalized by maximum possible size.

Because each country disaggregates total employment into different numbers of occupations, it is important to assess the impact of granularity of occupation classifications on our results. We do this by isolating the effects of different employment aggregation schemes within a single country. Here we use US data, where employment data is aggregated at several different hierarchical levels. While US employment data is typically tallied at the 6-digit occupational code, we aggregated employment additionally at the 5-digit code level and the 4-digit code level. We then recalculated the network size and density for all US cities under these alternative aggregation schemes. Results showed a consistent super-linear relationship regardless of employment aggregation level and showed no qualitative differences in scaling exponent (Fig 3).

**Fig 4:**
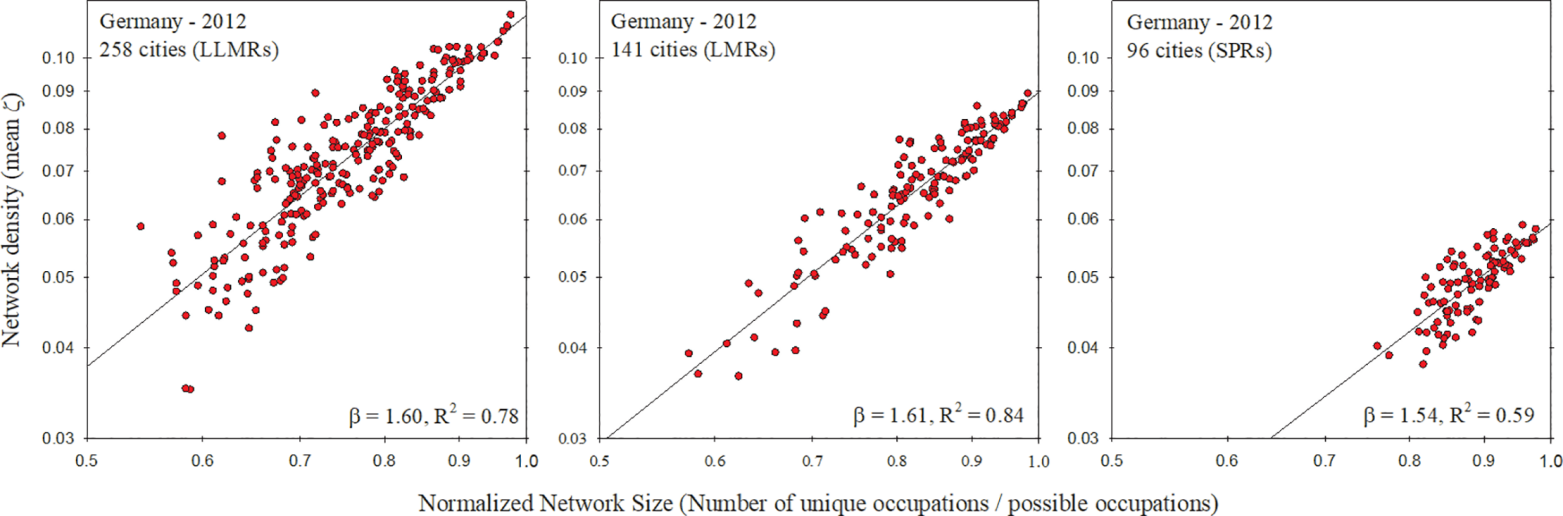
Occupational network size vs. density for German cities at different spatial aggregation levels. When the size of an occupational network is compared to its density, the resulting scaling exponents differed little when German employment is geographically aggregated into 258 LLMRs, 141 LMRs, or 96 SPRs. Note that, for comparability, network size has been normalized by maximum possible size.

Finally, we examined how the relationship between a city’s occupational network size and density is affected by the way in which a city is spatially defined. Each country has its own method of defining the spatial boundaries of its metropolitan areas, and so it is prudent to understand how the method of spatial demarcation affects our results. To isolate the effects of spatial delineation methodology, we use employment data from Germany, which has three alternative schemes for spatially defining its metropolitan areas. While our initial analysis used an aggregation method that results in 141 German metropolitan areas, we additionally analyzed employment data using two other aggregation methods that result in 96 urban units and 258 urban units, respectively. Keeping the German occupational classifications constant, we recalculated network size and density using these alternative spatial definitions. Results revealed a consistent superlinear relationship regardless of how metropolitan areas were defined, while showing no qualitative differences in scaling exponent (Fig 4).

## Discussion

### Superlinear scaling of network connectivity with network size

Most straightforwardly, the results presented here indicate that as the number of distinct occupation present in an urban area increase, the linkages among these occupations increase as well (on average). Obviously some of the connections among occupations result from complementarities as most occupations do not produce end products or services by themselves. But because the increase is greater than proportional, it is evidence that these connections are not infrastructural in nature, as is the case with other types of urban networks, nor is the formation of the linkages animated by economies of scale [40]. The superlinear scaling exhibited by urban occupational networks suggests instead increasing returns due to information aggregation [6].

The increase in network density is largely driven by two phenomena. The first is the appearance of rare, but highly interdependent pairs of occupations in larger networks. These highly specialized occupations tend to appear only in larger cities, both in terms of population and network size, that are able accommodate the prerequisite and complimentary occupations needed for these rare occupations. The second driver of increasing network density is that the proportion of occupations with low or negative link values with other occupations tend to decrease as a city’s network size increases. This is likely due to cities becoming more integrated, interdependent, and economically efficient as they increase in size.

### Differences among national urban occupational networks

The superlinear scaling of density versus size is exhibited by urban occupational networks corresponding to urban systems of widely differing vintage, history, socioeconomic development and technological capabilities. One can be confident that the superlinearity is therefore not an artifact. Nevertheless, a prominent feature of the result presented in Table 1 is the difference in scaling exponents among national urban systems. One possible reason for the highest scaling exponent occurring in the U.S. is that the U.S. occupational reporting scheme has the highest granularity, recognizing 812 unique occupations, while Germany recognizes 700, Canada 500, Australia 475, Sweden 355, and Portugal 641. However, results of our analysis on multiple aggregation levels of U.S. data (Fig 3) revealed little difference in scaling exponent across various levels of occupational aggregation. This suggests that differences in scaling exponent between countries is likely due to factors other than simply the number of unique occupations that each country recognizes (see Table 2 for full regression details).

Another possible reason for the difference in scaling exponent among countries is that there exists no standard method among countries for defining metropolitan areas. Even within a given country there are typically multiple hierarchical levels of spatial aggregation or alternative schemes of urban delineation. Yet, our results using these three spatial aggregation methods for Germany, presented in Fig 4, reveal little difference in scaling exponents. This suggests that differences in scaling exponent between countries may not be due simply to differences in how countries spatially define their metropolitan areas (see Table 2 for full regression details).

Yet another possibility for differences among countries’ scaling exponents is number and distribution of cities by size, where size is measured as the number of total employees. While the U.S. has 23 cities with at least 1 million employees, the smallest country in our study, Sweden, has only one. In addition, neither Canada nor Sweden have cities that compare in magnitude to the largest U.S. cities. While Sweden’s largest labor market has 1.3 million employees (Stockholm), Germany’s largest has 2.5 million (Berlin), and Canada’s largest has 2.8 million (Toronto), the largest U.S. labor market has 8.3 million employees (New York) and three other U.S. labor markets have 3 million or more. Thus, the scaling exponent of the U.S. may be influenced by the incomparable size and quantity of its largest cities.

## Materials and methods

### Data

Our analysis is based on employment statistics that aggregate the number of workers in each occupation in each metropolitan area of an entire country. In the current analysis, we use such datasets for six countries–the U.S., Canada, Germany, Sweden, Australia, and Portugal. While there are nuances to the way that each country defines its occupations, in general occupations are defined based partly what work is performed and partly on the skills and training needed to perform the work [41–43].

The spatial units of analysis for the U.S. urban system are its Metropolitan Statistical Areas (MSAs) for which employment data is compiled by the U.S. Bureau of Labor Statistics (BLS). MSAs consist of a core county, or counties, containing a city with a population of at least 50,000, plus adjacent counties having a high degree of social and economic integration with the core as measured through commuting ties. MSAs are unified labor markets and encompass geographical areas of high economic cohesion [44, 45]. The BLS included 380 MSAs in its 2013 employment data. Of those eight were excluded because they are in Puerto Rico, which has a unique economic environment, and two were excluded because, although they are treated as metropolitan areas by the BLS, they are classified as micropolitan areas by the U.S. Census Bureau. Together the remaining 370 U.S. metropolitan areas account for nearly 85% of U.S. population and over 90% of U.S. economic output. We use data from the BLS’s 2013 Occupational Employment Survey, which includes the estimated number of people employed in each of 812 distinct occupations for each MSA [46].

For the analysis of Canada’s urban system, the spatial units of analysis are the 32 Census Metropolitan Areas, which have a population greater than 100,000, and the 115 Census Agglomerations, which have a population between 10,000 and 100,000 [47]. These 147 units are defined by a high level of economic cohesion and are typically geographically contiguous. Employment data was extracted from Canada’s 2011 National Household Survey (NHS), which was compiled by Statistics Canada in conjunction with Canada’s quinquennial census. For each urban area, the survey collects the number of workers employed in each of 500 occupations.

For Germany the units of analysis are 141 Labor Market Regions (LMRs) as defined by Kosfeld and Werner [48]. LMRs consist of one or more of the 402 German districts. LMRs are characterized as essentially independent economic areas with close commuter links within the regional labor markets. The analyze the effects of alternate spatial definitions of metropolitan areas we apply two additional functional demarcations of regions available in Germany. First we use 258 Local Labor Market Regions (LLMRs) delineated by the German Federal Government for use by business development programs. Second, we make use of the 96 Spatial Planning Region*s* (SPR) used by the Federal Government for regional planning. LLMRs and SPRs are also based on accessibility and interdependence criteria such as the catchment areas and commuting flows but capture different geospatial definitions. Employment data is provided by the German Federal Employment Agency, which collects information on all employees subject to German social insurance contributions (including health, pension, long-term care, and unemployment insurance funds) as reported by employers. German employment data come from year 2012 and includes the number of workers in each of 700 occupations.

For Sweden the units of analysis are the 72 Functional Analysis Regions (FAs). Swedish FAs are delineated by the Swedish Agency for Growth Policy Analysis (Tillväxtanalys) and consist of one or more of the country’s 290 municipalities exhibiting a high level of commuting workers across municipal boundaries. Like U.S. MSAs, this transboundary movement of labor is taken to indicate a high level of economic cohesion [49]. Many Swedish FAs consist of only one or two municipalities, especially in the northern part of the country where population density is low, while the Stockholm FA–the largest–is an agglomeration of 28 municipalities. Employment data were extracted from Statistics Sweden’s 2012 microdata, which covers all individuals in the country’s Labor Force Survey and tabulates the number of employees in each of 355 occupations across the country’s municipalities.

For Portugal we use 23 metropolitan areas defined by Nomenclature of Territorial Units for Statistics level 3 (NUTS3). These areas are contiguous and stable regions that reflect urban socio-economic and administrative geographical unity. We exclude from the analysis two Atlantic archipelagos, the Autonomous Regions of Açores and Madeira, due to their unique social-economic contexts. NUTS3 correspond to Areas Metropolitanas e Comunidades Intermunicipais, which are formed for regional planning purposes by the Portuguese Local Public Administration, aggregating the 308 Portuguese municipalities according to their geospatial interdependencies (labor mobility, urban economy and public administration links, etc). We use metropolitan level employment data for 641 occupations provided by the 2012 Quadros de Pessoal, the Portuguese Linked-Employer-Employee-Data. Quadros de Pessoal is collected annually by the Portuguese Ministry of Employment and covers all establishments’ employees subject to Portuguese social insurance contributions.

For Australia we use 101 Significant Urban Areas (SUA) as defined by the Australian Bureau of Statistics’ Australian Statistical Geography Standard. These units represent towns and cities with 10,000 or more residents in either a single or a cluster of urban centers. The 2016 Australian census reports employment for SUAs in each of 475 occupations using the most recent Australian and New Zealand Standard Classification of Occupations (ANZSCO) at the 4-digit occupation codes level.

### Occupational interdependence

Two aggregate metrics are calculated for each city’s occupational interdependence network, the network’s size and its average connectivity (or density). In these networks, nodes are occupations and the weight of a link between any two nodes is a function of how often those two occupations are co-located in the same city. All urban economies within a national system have certain economic activities in common, namely those that address the needs of individuals and households and that satisfy demands common across a society. But what distinguishes urban economies from each other are those activities in which each city specializes. In calculating the occupational interdependencies that define an urban economic structure we focus on those occupations in which a city specializes, that is, those occupations that define a city’s comparative human capital advantage.

A city is specialized in an occupation if the proportion of the city's labor force engaged in that occupation is greater than the same proportion nationally. Thus, specialization can be stated in terms of the widely-used location quotient (*LQ*), which for occupation *i* in MSA *m* is defined as:

$$LQ_{i}^{\left(m\right)}=\frac{\left(x_{i}^{\left(m\right)}/\sum_{i}x_{i}^{\left(m\right)}\right)}{\left(\sum_{m}x_{i}^{\left(m\right)}/\sum_{m}\sum_{i}x_{i}^{\left(m\right)}\right)},$$ (2)

where *x*_*i*_^(*m*)^ is the number of workers employed in occupation *i* in city *m*. City *m* is specialized in occupation *i* if its location quotient *LQ*_*i*_^(*m*)^ > 1. Thus, for each country in our study we derive an *M* × *O* specialization matrix where *O* is the number of recognized occupations in a country, *M* is the number metropolitan areas in a country (for which employment data exists), and element *a*_*mi*_ = 1 if *LQ*_*i*_^(*m*)^ > 1 and 0 otherwise.

How can one infer from the presence of specialized occupations in an MSA that their co-location is not merely accidental but indicative of interactions through which complementarities are realized and information flows? We employ conditional probability: specifically, in this context if the presence of one specialized occupation in an MSA is statistically affected by the presence of another specialized occupation, one would expect conditional probabilities to differ from marginal ones. The co-location patterns of specialized occupations among all cities to define the interdependence between any two occupations *i* and *j*, *ζ*_*ij*_, as:

$$\zeta_{ij}=\frac{P\left[LQ_{i}^{\left(m\right)}>1,LQ_{j}^{\left(m\right)}>1\right]}{P\left[LQ_{i}^{\left(m'\right)}>1\right]P\left[LQ_{j}^{\left(m''\right)}>1\right]}-1,$$ (3)

where *m*, *m'*, and *m''* denote a randomly selected city [21]. This metric measures how an MSA's specialization in one occupation may enhance or hinder its specialization in another. The emphasis on “may” acknowledges that—as is the case for many statistical analyses—without additional information or experiments, our analysis cannot imply direct causality; at best, it identifies structural relationships and points to potential places where one may search for such causality. Thus, *ζ* has the characteristic of being positive when two occupations co-occur in a city more frequently than expected by chance, and of being negative when they co-occur less frequently. Note that, because our networks are undirected, the interdependence is symmetric so that *ζ*_*ij*_ = *ζ*_*ji*_. The calculation of *ζ* is specific to each nation’s occupational classification system but applies to every city within that nation.

We treat the occupational interdependencies to be the weights that link every pair of occupations in an occupational interdependence network. Network weights are a quantification of the intensity of the link between any two nodes in a network. Weights could indicate the magnitude of flows between nodes, the frequency of interaction, the strength of a relationship, etc. In our occupational networks, weights quantify the intensity of co-occurrence for any two occupations.

Because of heterogeneity among occupational relationships, occupations are not uniformly distributed within a country’s occupational interdependence network. Instead, a country’s network typically contains a denser core of highly interdependent occupations and a periphery of occupations that tend to be weakly or negatively interdependent with others. Two occupations *i* and *j* tend to be, on average, close to each other in a country’s occupation network if *ζ*_*ij*_ is positive and farther apart if *ζ*_*ij*_ is negative. Thus, occupations that appear closer in a network also tend to co-exist within a given city more frequently.

Having specified an occupation network specific to each country, we can then locate specific cities within a country’s network space. To understand what is meant by a city’s location in a network, it is important to note that no city in our study has employees in every possible occupation. Instead, each city contains a subset of all possible occupations and when this subset is mapped to nodes within the full occupational network, it defines a *subnetwork* representative of the city, which we equate here with the city’s location within the full network.

Note that for Germany, we used employment data at the aggregation level of the 141 LMRs to calculate the country’s zeta values. Furthermore, while 10 MSAs are excluded from the US comparative analysis (8 because they were in Puerto Rico, 2 because they are generally recognized as micropolitan statistical areas), employment for those 10 were included in the basis for calculation US zeta values to ensure that interdependencies were based on the most comprehensive data possible.

### Density and size of urban occupational networks

Given these weighted occupational networks for metropolitan areas in our countries of interest, we seek to determine the relationship between each network’s average connectivity and its size. Here we equate a network’s average connectivity with the network’s density. The traditional metric of network density, which applies to unweighted networks, is simply the number of links in a network divided by the number of possible links [50]. Because our networks are weighted, we utilize a subsequently developed generalized definition of network density, which is the sum of all weights divided by the number of possible links [51, 52]. Thus, the density of a weighted network is synonymous with the average of all weights in that network.

An unweighted and undirected network of *N* nodes can be represented as a symmetric *N* × *N* matrix in which element *a*_ij_ = *a*_ji_ = 1 if and only if a link exists between nodes *i* and *j*. Otherwise *a*_ij_ = *a*_ji_ = 0. Typically, an arbitrary threshold of interaction strength between two nodes is used to determine the existence of a link (e.g. *a*_ij_ = 1), and once determined, all links are thus equivalent. Such networks are amenable to wider range of analytical tools and are typically easier to analyze. However, in collapsing interaction strength to a binary determination, important information regarding the network and the system it governs is lost [53]. Thus, weighted networks exist as an alternative representation of interaction systems in which the elements of the network’s adjacency matrix may be other than 0 or 1. Instead, each element holds a weight *w*, or a value representative of the strength of interaction. In an undirected network weights are symmetric so that *a*_ij_ = *a*_ji_ = *w*_ij_.

For a given city *m*, the generalized density *D*^*m*^ of its occupational network can be calculated as:

$$D^{m}=\frac{2}{N^{m}(N^{m}-1)}\sum_{i<j}^{N^{m}}\zeta_{ij},$$ (4)

where *N*^*m*^ = the number of nodes (e.g., unique occupations) in city *m* and *ζ*_*ij*_ is the interdependence (e.g., weight) between occupations *i* and *j*, both of which must be present in *m*. Because our occupational networks have an interdependence value for every pair of occupations, they are complete networks (e.g. every node is linked to every other node) and their density is therefore the mean *ζ* across all links in a given city. Note that when equation 4 is applied to an unweighted network, so that *ζ*_*ij*_ = 1 when a link exists between nodes *i* and *j* and *ζ*_*ij*_ = 0 when there is no link, *D* ∈ [0, 1] and is simply the number of links present divided by the number of links possible.

A noteworthy aspect of our networks is the existence of negative weights, which is being increasingly addressed in analyses of networks [54–56]. By permitting negative weights, we incorporate into our analysis those instances where two occupations interact negatively. That is, only one of the pair tends to exist in a city, suggesting a form of competitive exclusion or similar interference.
